# Investigating plantar soft tissue elasticity modulation using air insoles during walking

**DOI:** 10.1186/s12891-025-09427-9

**Published:** 2026-01-08

**Authors:** Ardha Ardea Prisilla, Fahni Haris, Yih-Kuen Jan, Ben-Yi Liau, Wei-Cheng Shen, Chang-Wei Hsieh, Wen-Hung Chao, Chun-Ming Lien, Chi-Wen Lung

**Affiliations:** 1 Department of Fashion Design, Sekolah Tinggi Desain LaSalle Jakarta, Jakarta, 10220 Indonesia; 2 Department of Digital Media Design, Asia University, Taichung, 413305 Taiwan; 3 School of Nursing, Universitas Muhammadiyah Yogyakarta, Yogyakarta, 55183 Indonesia; 4 Rehabilitation Engineering Lab, Department of Health and Kinesiology, University of Illinois at Urbana-Champaign, Champaign, IL 61820 USA; 5 Department of Automatic Control Engineering, Feng Chia University, Taichung, 407102 Taiwan; 6 Department of Creative Product Design, Asia University, Taichung, 413305 Taiwan; 7 Department of Electrical Engineering, National Dong Hwa University, Hualien, 974301 Taiwan; 8 Department of Culture Creativity and Digital Marketing, National United University, Miaoli, 360302 Taiwan; 9 Department of Commercial Design and Management, National Taipei University of Business, Taoyuan, 32462 Taiwan

**Keywords:** Diabetic foot ulcers, First metatarsal head, Insole stiffness, Plantar stiffness, Walking duration

## Abstract

**Background:**

In the forefoot region, diabetic foot ulcer (DFU) is more likely to develop due to elevated plantar pressure and repetitive loading. This study investigated the effects of different forefoot insole pressures and walking durations on the elasticity of the plantar tissue to identify conditions that may help prevent DFU.

**Methods:**

With thirteen healthy participants, we conducted a controlled walking exercise at a speed of 3.6 mph on a treadmill, comparing three different inner pressures of 80, 160, and 240 mmHg with two different walking durations of 10 and 20 min. We measured the elasticity of the first metatarsal head (M1) using a motor-driven indentation system with ultrasound post-walking exercise. We measured the effective Young’s modulus (*E*) for analysis, where *E1* is taken from 5% of the initial thickness of the Toe region’s elasticity, *E2* is taken from 10% of the initial thickness of the Heel region’s elasticity, and *E3* is taken from 15% of the initial thickness of the Linear region’s elasticity. ANOVA and paired t-test analyses are used to find the relation between durations in the post-walking conditions.

**Results:**

The effect of the different insole hardnesses using ANOVA indicated that the 240 mmHg insole hardness significantly reduced the stiffness of M1 in *E1* (*P* = 0.042) and *E2* (*P* = 0.046), but not in *E3* (*P* = 0.059). In the paired t-test, no significant differences were found between the 10 and 20 min walking durations for the three insole hardnesses.

**Conclusion:**

The study concluded that after a 10-minute walking duration, using 240 mmHg insole hardness has lower stiffness than the recommended stiffness range. These findings highlight the importance of optimizing insole pressure and walking duration in maintaining foot health, and provide evidence to guide the design of functional air insoles to reduce the risk of DFU formation.

**Trial registration:**

ClinicalTrials.gov Identifier: NCT06746597. Registered on 2024-12-09 (retrospectively registered).

## Highlight

1. A significant change was observed in the toe (initial) and heel (middle) regions of the J-shaped curve, reflecting variations in Young’s modulus across different insole pressures (80, 160, and 240 mmHg) and walking durations (10 and 20 min) at the first metatarsal head (M1).

2. The 240 mmHg insole hardness contributed to the lowest measurement of M1 in *E1* (toe 5%) and *E2* (heel 10%), but not in *E3* (linear 15%) after walking for 10 min.

3. There was no significant difference in plantar tissue material property changes in M1 using 80 mmHg, 160 mmHg, and 240 mmHg inner pressure of the insole between the 10 and 20 min walking duration.

## Introduction

Diabetes mellitus (DM) is a multifactorial disorder that affects nearly all body tissues and is characterized by elevated blood glucose levels that exceed a specific threshold [1]. DM is a major public health concern worldwide and is associated with high mortality, morbidity, and disability [2]. A report from the International Diabetes Federation stated that the prevalence of DM reached 537 million adults aged 20–79 years in 2021, and it is estimated that it will reach 643 million by 2030 [3]. There is an expected increase in expenditures related to diabetes from US$ 966 billion in 2021 to US$ 1054 billion by 2045 [2]. DM can greatly impact health and social insurance systems; therefore, policymakers should take immediate action to address this issue [4].

Exercise is a preventive intervention that may promote health and reduce the risk of chronic complications in patients with DM [5, 6]. Exercise, such as walking, is the most common form of physical activity that patients with DM can engage in [7, 8]. According to Wu et al., walking for 10 min at 9 km/h and 20 min at 6 km/h significantly increases the ratio of wavelet amplitudes associated with metabolic, myogenic, respiratory, and cardiac factors [9]. Several studies have demonstrated that walking is an effective intervention for patients with DM to manage their blood sugar levels [6, 10]. Additionally, a study by Kasmad et al. found that 78.6% of respondents reported a decrease in blood sugar levels after brisk walking compared to the control group [11]. However, patients with DM experience increased stiffness compared to healthy individuals, and stiffened skin may break down more easily, especially under repetitively high vertical and shear forces on the foot, such as during walking [12, 13]. The pathophysiological process during walking may alter the foot structure and cause deformity, contributing to changes in stiffness. A study by Çakici et al. reported that patients with DM have a higher level of plantar stiffness than healthy people [14].

Plantar tissue has a complex microstructural composition, and different compressive strain rates can modify this structure, thereby affecting its elasticity [15]. Normally, the stress-strain curve of collagenous tissues in the plantar tissue is J-shaped and is usually divided into three sections: *E1* (toe), *E2* (heel), and *E3* (linear) [16]. In *E1*, the plantar tissue is relatively soft, and many of the plantar tissue’s structural responses are driven by elastin because collagen fibers are slack and non-load-bearing [15]. In *E2*, elastic fibers begin to stretch and realign in the direction of the applied force, and collagen in the gap sections begins to resist deformation as the strain-stress curve progresses. In *E3*, collagen fibrils are already realigned, causing deformation owing to the sliding of elastin or collagen [16, 17]. Increased elasticity in the plantar region affects the walking performance [9].

Previous studies have shown that the elasticity and stiffness of plantar tissue provide additional valuable information for detecting tissue alterations in patients with DM [12, 18]. Various methods have been developed to measure the elasticity of soft tissues in patients with DM [18, 19]. In recent studies, the use of insoles with appropriate hardness has been found to enhance elasticity, mitigate discomfort, and decrease the risk of diabetic foot ulcer (DFU) by evenly distributing pressure across the feet [5, 13]. Significant progress has been made in the development of air insoles related to their hardness over the past few years, with encouraging results [20]. An air insole encloses air within a flexible bag inside a shoe, increasing shock absorption, and providing superior stability and comfort during ground contact [21, 22]. Growing evidence supports the idea that air insoles have multifaceted benefits, highlighting their advantages and facilitating more effective exercise regimens, particularly for individuals with DFU. Softer insoles have been noted for their effectiveness in reducing peak plantar pressure, thereby preventing injuries, such as metatarsal problems and blisters [23]. However, the plantar elasticity associated with the efficacy of insole hardness has yet to be analyzed.

Furthermore, longer walking durations are associated with an increased complexity index of plantar soft tissues, suggesting that the structure and functionality of these tissues may have been altered [24]. Studies have also revealed that repetitive loads are placed on plantar soft tissues with prolonged walking, causing elasticity to increase [25, 26]. However, the changes in plantar elasticity related to different walking durations have not yet been evaluated. Therefore, using suitable inner pressure and following an appropriate walking duration has the potential to reduce plantar stiffness.

This study investigated the effects of different insole inner pressures and walking durations on the elasticity of plantar tissue to determine their potential implications for reducing the risk of DFU. A comprehensive understanding of the complexity of how insole inner pressure interacts with walking duration to affect elasticity values would provide valuable insights into mechanistic prevention strategies for DFU. Therefore, the aim of this study was to determine the most suitable walking condition based on the effect of insole inner pressure and walking duration on the elasticity of the plantar tissue. A thorough understanding of the elastic properties of plantar tissues is essential for the development of interventions that can efficiently redistribute pressure from areas at high risk, thus minimizing the likelihood of tissue breakdown and ulceration. By understanding these properties, healthcare professionals can design preventive strategies and interventions to reduce DFU.

## Materials and methods

### Participants

Study design is according to CONSORT guidelines. A total of 13 healthy participants who could walk independently without any device were recruited in the study. The sample size was determined based on our previous studies examining plantar soft tissue properties under different insole conditions [5, 10]. Study participants were excluded if they had active ulcers on their feet, DM, pain in any lower extremity joint, or a history of foot amputation or other lower extremity surgeries. The healthy participants were selected to establish a controlled baseline of plantar tissue response, excluding the confounding effects of diabetes-related complications, such as neuropathy or vascular impairment [27]. The participants’ demographic details are presented in Table 1.

**Table 1:** Participant demographics and baseline characteristics

Variable	Mean±SD	Range
Age (years)	27.0±7.3	21–39
Body weight (kg)	56.0±7.9	43–68
Body height (cm)	165.8±8.4	153–178
Body Mass Index k(g/m^2^)	20.3±1.7	17.2–23.3

Sex (Male/Female): 7/6

There were several criteria for selecting the participants, including shoe size (EU size 41–43) for men, and shoe size (EU size 36–38) for women and a body weight of less than 80 kg with a dominant right leg. The studies involving human participants were approved by the Central Regional Research Ethics Committee of China Medical University, Taichung, Taiwan (CRREC-112-130), and were subsequently registered in the International Trial Registry [ClinicalTrials.gov: Identifier NCT06746597; https://clinicaltrials.gov/study/NCT06746597] with the first posted of Study Registration Dates on 2024-12-09. The studies were conducted under local legislation and institutional requirements. All participants provided written informed consent to participate in this study and were assured that their personal information would remain confidential. As required by the Declaration of Helsinki, respondents were able to withdraw from the study at any time, and their responses were anonymized. The recruitment period for participants began in June 2022 and ended in August 2022.

### Equipment

**Fig. 1:**
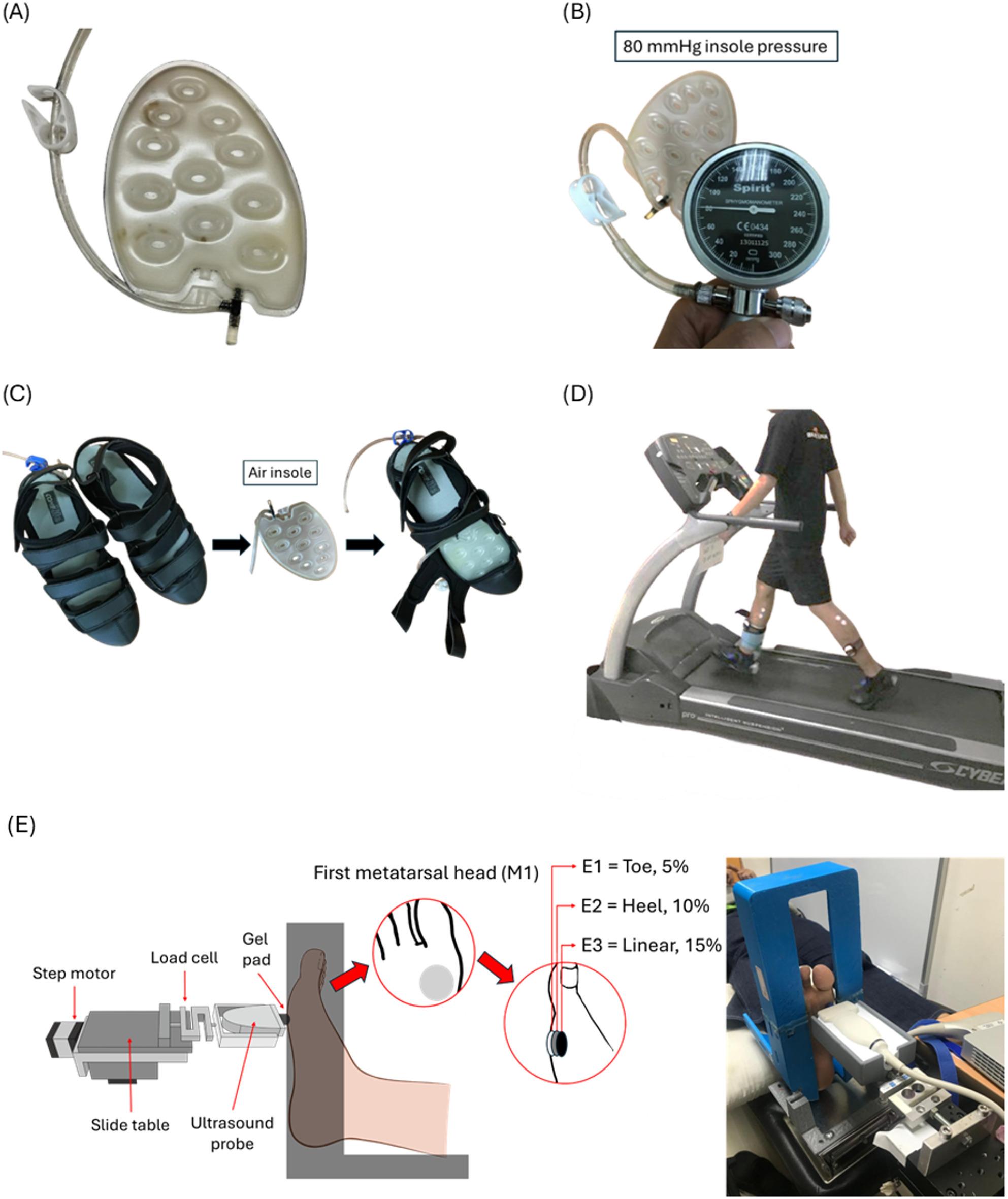
The device used in the research, (**A**) Thermoplastic Polyurethane insole, (**B**) Measuring the inner pressures of the insole (illustration for 80 mmHg inner pressure insole), (**C**) Shoes with inner pressure insole, (**D**) Subjects walked on a treadmill at constant speed with different insole hardnesses applied in the footwear. **E** Schematic diagram showing the indentation system. M1, first metatarsal head. *E1* = Toe 5%, *E2* = Heel 10%, *E3* = Linear 15%

In our earlier studies, we selected three insole hardnesses to ensure appropriate elasticity for walking in the insoles set at three different inner pressures (80, 160, and 240 mmHg) [5, 28]. The insoles were composed of thermoplastic polyurethane (Hsin He Hsin Co., Ltd., Taichung, Taiwan) (Fig. 1A and B). Our insole type is the forefoot insert, which covers the anterior part of the plantar fascia, focusing on the ball of the foot and the toes, as the region mostly affected by the DFU is in the metatarsal region [29]. Air insole hardness values were determined using a GS-701 N Shore durometer (Teclock Co., Ltd., Nagano, Japan) [30]. We ensured all measurements were conducted with the insole placed on a solid, flat surface to minimize deflection effects. The measurement approach aligns with standard practice in biomechanical studies assessing the Shore hardness of insole materials [31].

The present study measured three different hardnesses: an 80 mmHg hardness value at 51.7 ± 1.5 Shore, a 160 mmHg hardness value at 54.7 ± 0.6 Shore, and a 240 mmHg hardness value at 57.7 ± 0.6 Shore. Participants were requested to wear commercial footwear (Best Airwalk, BAW, Supreme Air, Hsin He Hsin Co., Ltd., Taichung, Taiwan) and the air insole designed for this study and were asked to walk on a treadmill (Cybex DE-20427 A, Cybex, Taoyuan, Taiwan) (Fig. 1C and D).

### Plantar elasticity

The elasticity of the first metatarsal head (M1) was determined using an indenter system that responds to pressure loading during the creep and stress relaxation processes [18, 32]. This system integrates ultrasound technology to monitor real-time soft tissue thickness and deformation using echo signals. An ultrasound transducer is aligned in series with a compressive load cell, enabling measurement of the force response generated during ultrasound wave transmission [33]. Each test involved five cycles of loading and unloading. Changes in the M1 tissue thickness were captured throughout the cycles, with the ultrasound echo signals used to determine both tissue thickness and deformation [18]. This system operates at a frequency of 12 MHz (5–12 MHz, 128 elements, 39 mm array footprint, Telemed, Vilnius, Lithuania) with a PC-based ultrasound system (ArtUs EXT-1 H scanners, Telemed, Vilnius, Lithuania) connected to a 49-N load cell (Model UKA-E-005, Li-Chen Measure Co., Ltd., Kaohsiung, Taiwan) in series for indentation of the soft tissues. The initial tissue thickness and force-deformation responses were determined using an ultrasonic signal [34].

**Fig. 2:**
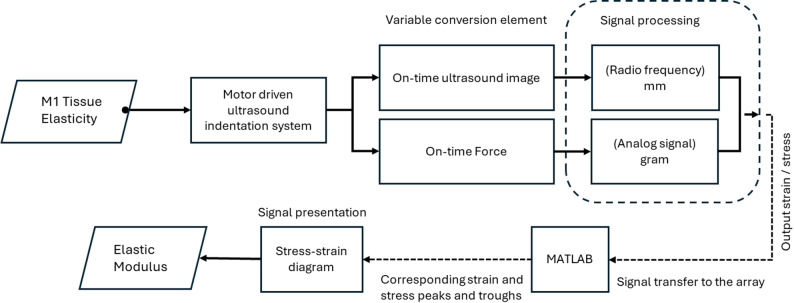
An illustration of a motor-driven ultrasound indentation measurement system

The present study recorded sampling rates of the image frames and force data using 22.5 Hz and 100 Hz DAQ data acquisition devices (USB-6218, National Instruments, Austin, TX, USA). The normal ultrasound sampling rate is 40 MHz, but each probe has some variation. The maximum frequency is 40 MHz. The deep image has a frequency of 20 MHz, and the thin image has a frequency of 40 MHz. As an alternative to manual indentation, a stepper motor was used in this indentation system (Model TL-SL1010-X, Tanlian Electro Optics Co., Ltd., Taoyuan, Taiwan) with a total travel of 50 mm and step travel of 0.000625 mm. The recorded tissue thickness was 4 mm, driven by a 1600 microstepper revolution (Model TL-1T, Tanlian Electro Optics Co., Ltd., Taoyuan, Taiwan) to achieve automated cyclic indentation. The ultrasound transducer probe was equipped with a standoff gel pad (coupling medium, cylinder with 9 mm diameter, 20 mm thickness, Aquaflex ultrasound gel pad, Parker Laboratory, Orange, NJ, USA) [18]. Figure 2 illustrates the motor-driven ultrasound indentation measurement system.

**Fig. 3:**
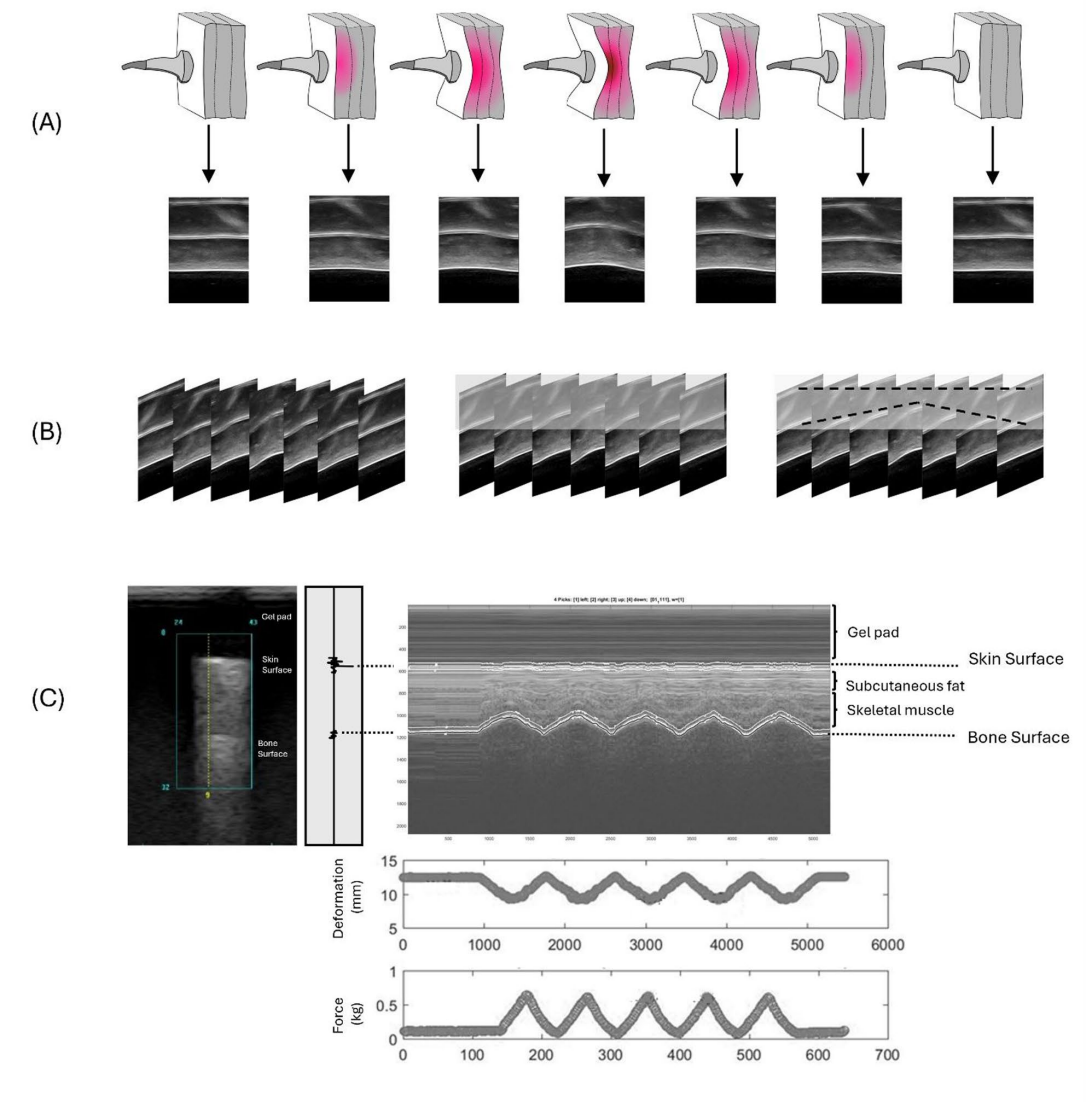
A conceptual diagram of the motorized ultrasound indentation system used for tissue compression. **A** Time-sequenced B-mode ultrasound image array. **B** Extraction of time-lapse changes in ultrasound beam data. **C** Transformation of soft tissue anchor points during compression with an example of indentation data from the M1 region. The M-mode ultrasound image displays two echoes: the first from the transducer–skin interface and the second from the tissue–bone interface. The distance between these echoes determines soft tissue thickness

During the experiment, the subjects were asked to lie supine on a hospital bed, with their feet secured in a holder (Fig. 1D). From real-time ultrasound images, an indent compression of 20% of the total subcutaneous soft tissue thickness was determined after a preload of less than 0.5 N was applied over the M1 perpendicularly [33, 35]. The 20% strain was determined to ensure both physiological relevance and experimental stability, while previous study [36] have shown that plantar soft tissues may approach incompressibility at a strain of approximately 40%. A study found that the indentation rate ranged from 0.5 to 1 mm/s, with a peak load of about 5 N, resulting in approximately 20% deformation of the full tissue layer [33]. Another study, utilizing Digital Image Correlation to analyze strain on the plantar surface, found that principal strains at the metatarsal heads during the stance phase ranged between 10% and 20%. This indicates that a 20% strain level aligns with the typical mechanical demands experienced by plantar tissues during normal gait [37]. Cyclic loading of 40 s was applied at intervals of approximately 8 s per cycle [34]. Figure 3 illustrates A conceptual diagram of the motorized ultrasound indentation system used for tissue compression. Before testing, variables related to strain were adjusted according to the characteristics of each subject. The stress can be calculated as the area of contact between the probe and M1, which is fixed after the load cell has recorded the force [33]. A displacement rate of 0.5–2 mm per 1 s, and the response force was 400–600 g.

### Experimental procedures

In this study, participants randomly selected their inner pressure insoles and walking duration. A trained nurse specialized in wound care conducted the intervention configurations. Before the walking intervention, participants and the trained nurse were informed of six different walking conditions. These were completed over a maximum of six days (one session per day) or a minimum of three days (two sessions per day). Each participant was tested with three different insole pressure settings and two walking durations. Insoles were placed inside the shoes in the metatarsal and toe regions.

The participants were instructed to remove their socks and shoes and lie down in a supine position for 20 min to minimize the impact of prior weight-bearing activities on muscle fatigue and plantar pressure. On days with two walking sessions, a 20 min washout period was implemented between tests to minimize potential carryover effects. This protocol was based on evidence from [38], which demonstrated that a 20 min rest is sufficient to restore neuromuscular function after moderate physical activity. Three different air insole pressures (80, 160, and 240 mmHg) and two walking durations (10 and 20 min) were used in this study. Each testing session lasted approximately one hour, including a 20 min rest period and a walking phase of either 10–20 min, as well as elasticity measurements before and after walking that took approximately 15 min each. Six walking protocols have been summarized in Table 2.

**Table 2:** Experiment walking condition

Random Configurations	Walking Condition
1	80 mmHg, 10 min
2	80 mmHg, 20 min
3	160 mmHg, 10 min
4	160 mmHg, 20 min
5	240 mmHg, 10 min
6	240 mmHg, 20 min

All 13 randomized participants completed all six intervention configurations, with each participant serving as their own control. Participants were monitored for discomfort throughout the experiment, and no adverse events were observed. Interim analyses and stopping guidelines were not applicable to this study. No additional or concomitant care was provided to participants in any group during the trial.

Following the American Guidelines and American Diabetes Association, 3.6 mph was selected as the recommended walking speed [39, 40]. The present study was part of a larger project investigating the effects of different inner pressure insoles and walking durations on the properties of the soft tissues in the plantar region [5]. The elasticity was measured before and after the walking period. The data from before walking was served as the baseline, following a standardized rest period to ensure consistent tissue conditions [5, 10, 41]. The elasticity assessments were conducted immediately, in less than 5 min after each walking trial, to capture the post-exercise mechanical response of the plantar tissues. Following our previous studies, we evaluate a composite plantar soft tissue layer between the skin surface and M1 [5, 8, 10, 41].

### Data analysis

Traditionally, the Effective Young’s modulus (*E*) has been used to quantify the elastic properties of soft tissues [33, 35]. A study by Egorov suggested that some tissue types exhibit nonlinear stress-strain behavior when subjected to mechanical testing. However, to simplify their analysis, the stiffness of these tissues can be represented by an Effective Young’s modulus. This Effective Young’s modulus is derived from a linear relationship between two specific points on the stress-strain curve, essentially approximating the overall tissue behavior linearly over a particular range of deformation [42]. Therefore, the Effective Young’s modulus may be used to assess whether the soft tissue structure has changed after the walking intervention. Based on a study by Ayyildiz et al., we segmented the data and applied different compressive strain rates [43]. In our study, the compression rate was displacement-controlled and kept within a 0.5–2 mm range over 1 s. Although tissue thickness varied slightly across insole regions, the same displacement rate was applied uniformly to all regions to ensure consistency. The equation used to extract *E* is as follows:

1 $$\:\boldsymbol{E}=\frac{\left(1-{\mathbf{v}}^{2}\right)}{2\mathbf{a}\:\mathbf{k}\left(\mathbf{v},\frac{\mathbf{a}}{\mathbf{h}}\right)}\:\frac{\mathbf{P}}{\mathbf{w}}$$

where v is Poisson’s ratio, a is the radius of the indenter, k is a scaling factor dependent on Poisson’s ratio, the radius of the indenter, and tissue thickness, h is the thickness of the soft tissue, P is the force of pressure loading (indentation), and w is the depth of indentation. Poisson’s ratio for biological soft tissues is generally 0.45. The radius of the indenter, which is the ultrasound transducer, is 4.5 mm [33, 43]. The k value was derived from Hayes et al. [35]. A MATLAB R2020b (MathWorks Inc., MA, US) image processing program was used to convert the ultrasound values and pressure into an effective Young’s modulus *E* for analysis.

**Fig. 4:**
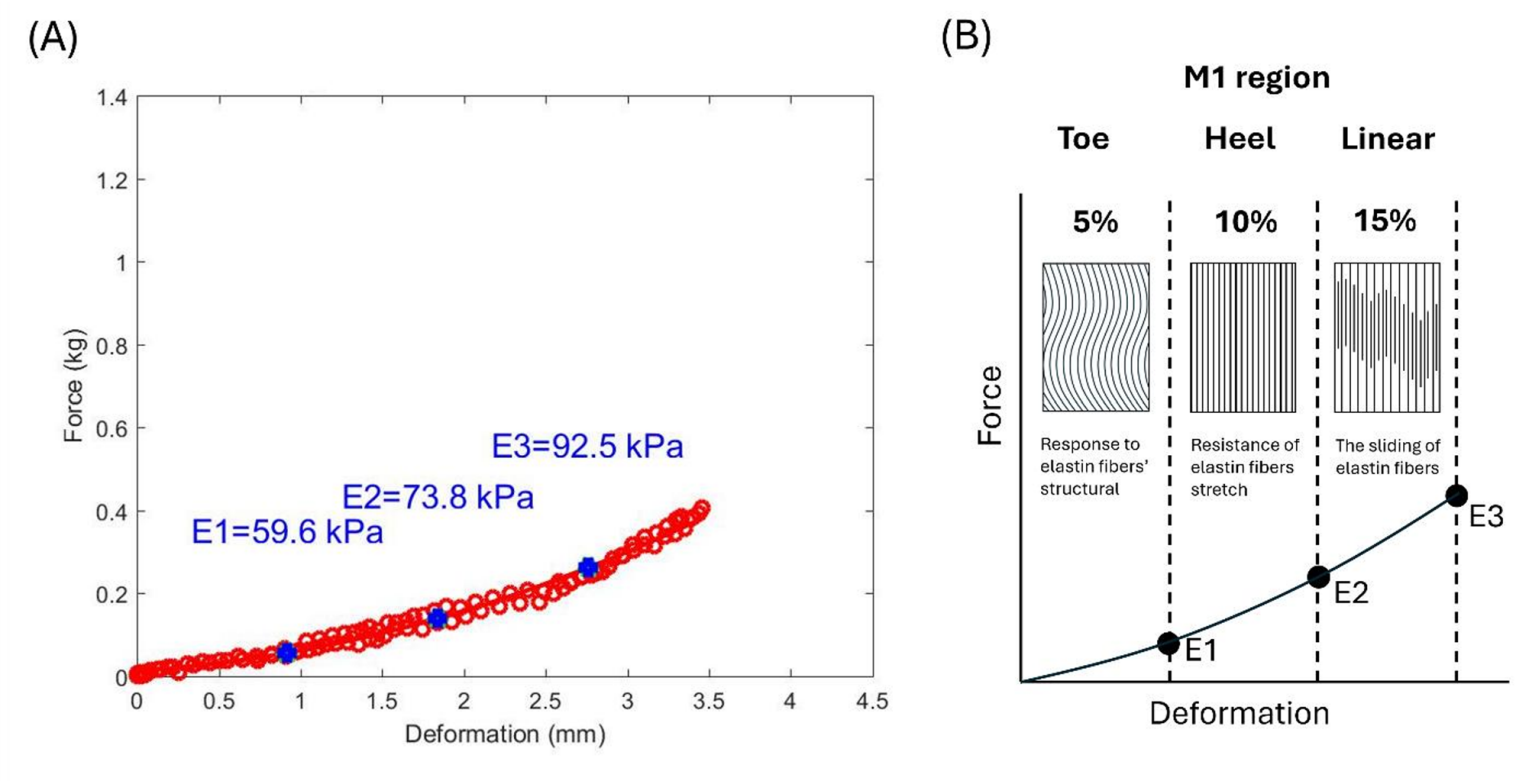
The process of identifying the elasticity value on the M1. **A** Deformation with force shows the elasticity; (**B**) Compressive stress-strain behavior of the M1. *E1* = 5% of the initial tissue thickness of the Toe regions’s elasticity, *E2* = 10% of the initial tissue thickness of the Heel regions’ elasticity, *E3* = 15% of the initial tissue thickness of the Liner regions’ elasticity. M1 = first metatarsal head

Figure 4A illustrates how the mechanical response of skin tissue is highly nonlinear owing to its microstructural composition [15]. Figure 4B illustrates the compression stress-strain curve of the skin of collagenous tissues, related to the force and deformation phase. In the toe and heel regions, fibers undergo buckling and bending along with minimal recruitment and straightening. Most fiber recruitment and alignment take place in the linear region up to the critical load, after which fibril sliding and stretching occur [16, 44]. We classified them into Toe (*E1*), Heel (*E2*), and Linear (*E3*) Sects [16, 17]. *E1* was approximately 5% of the initial tissue thickness of the Toe region’s elasticity, *E2* was approximately 10% of the initial tissue thickness of the Heel region’s elasticity, and *E3* was approximately 15% of the initial tissue thickness of the Linear region’s elasticity [33, 34].

### Statistical analysis

Elasticity values are presented as mean ± standard error. In this study, the main effect of the inner pressure insole and walking duration, as well as the interaction effect between the inner pressure insole and walking duration, were analyzed using a One-way analysis of variance (ANOVA) with the least significant difference (LSD) post-hoc test, we conducted pairwise comparisons of elasticity between the three inner pressures of the insoles and two walking durations [5, 25]. A paired t-test was used to determine whether there were differences in elasticity values between the inner pressure of the insole and the walking duration. After trial commencement, the planned multivariate analysis was omitted due to the limited sample size. No changes were made to the outcome measures. All participants (*n* = 13) were included in the analysis, and there were no missing data. SPSS version 22 (IBM, NY, USA) was used for all statistical analyses with a significance level of 0.05. We focused on analyzing the post-intervention measurements. This approach enabled us to specifically assess how different insole pressures influenced tissue behavior after walking, under a consistent and fatigued condition [41].

## Results

### Effect of inner pressures of insoles

**Fig. 5:**
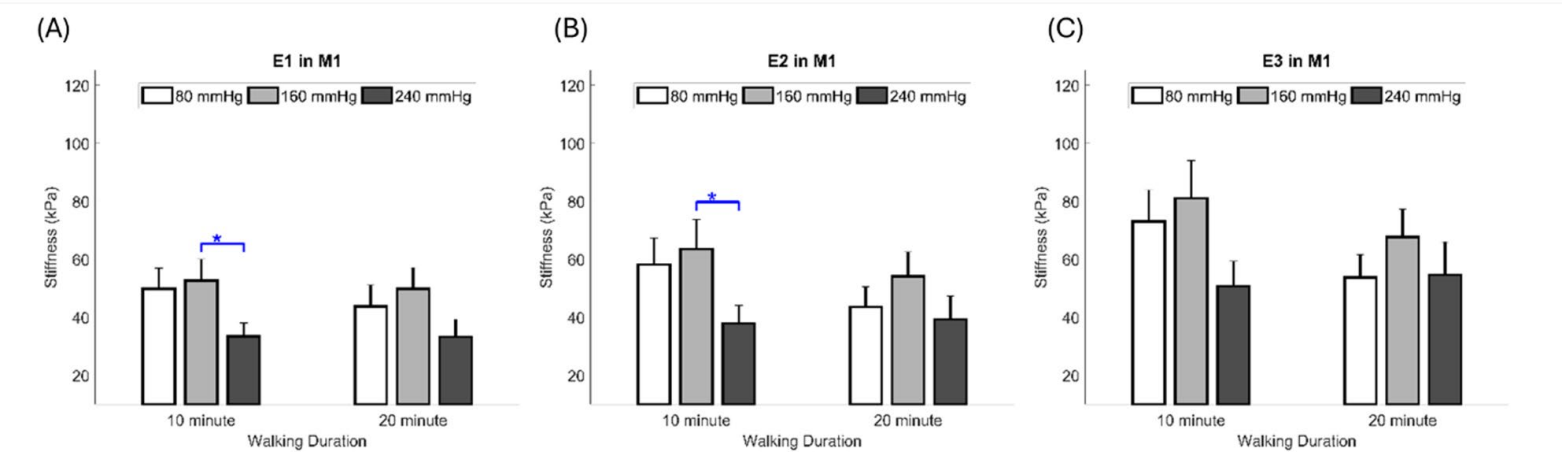
Comparisons of the effect of different insole hardnesses on the elasticity at two walking durations. **A** Effect of the insole hardness on the elasticity in *E1*; (**B**) Effect of the insole hardness on the elasticity in *E2*; (**C**) Effect of the insole hardness on the elasticity in *E3*. Data are shown as mean ± standard errors. * a significant difference (*P* < 0.05). M1 = first metatarsal head. *E1* = Toe 5%, *E2* = Heel 10%, *E3* = Linear 15%

Data were analyzed from all 13 participants, and One-way ANOVA results showed that the insole hardnesses were significantly different in M1 tissue when walking for 10 min. The first result in *E1*, 240 mmHg insole hardness, has significantly lower stiffness (higher elasticity) than 160 mmHg (33.4 ± 4.6 vs. 52.7 ± 7.3 kPa, *P* = 0.042) (Table 3; Fig. 5A); the second result in *E2*, 240 mmHg insole hardness was significantly lower stiffness than 160 mmHg (37.9 ± 6.3 vs. 63.5 ± 10.3 kPa, *P* = 0.046) (Table 1; Fig. 4B). However, *E3* did not show any significant differences (Fig. 4C).

**Table 3:** Effect of insole hardness on elasticity

Region	Walking Duration	Inner Pressure of Insole			One-way	LSD		
					ANOVA	Post hoc		
		80 mmHg (Mean ± SE)	160 mmHg (Mean ± SE)	240 mmHg (Mean ± SE)	*P* value	80 mmHg vs. 160 mmHg	80 mmHg vs. 240 mmHg	160 mmHg vs. 240 mmHg
*E1 *(kPa)	10	49.8±7.2	52.7±7.3	33.4±4.6	0.090	0.754	0.082	0.042*
	20	43.8±7.3	49.8±7.3	33.2±6.1	0.244	0.543	0.288	0.099
*E2* (kPa)	10	58.2±9.2	63.5±10.3	37.9±6.3	0.107	0.676	0.109	0.046*
	20	43.6±7.0	54.1±8.4	39.5±7.9	0.401	0.346	0.713	0.193
*E3* (kPa)	10	73.0±10.9	81.1±13.0	50.8±8.5	0.144	0.604	0.162	0.059
	20	53.7±8.0	67.7±9.6	54.7±11.3	0.533	0.317	0.943	0.353

Data are presented as the mean ± standard error

*E1* = Toe 5%, *E2* = Heel 10%, *E3* = Linear 15%

* Significant difference (*P* < 0.05)

### Effect of walking duration

**Fig. 6:**
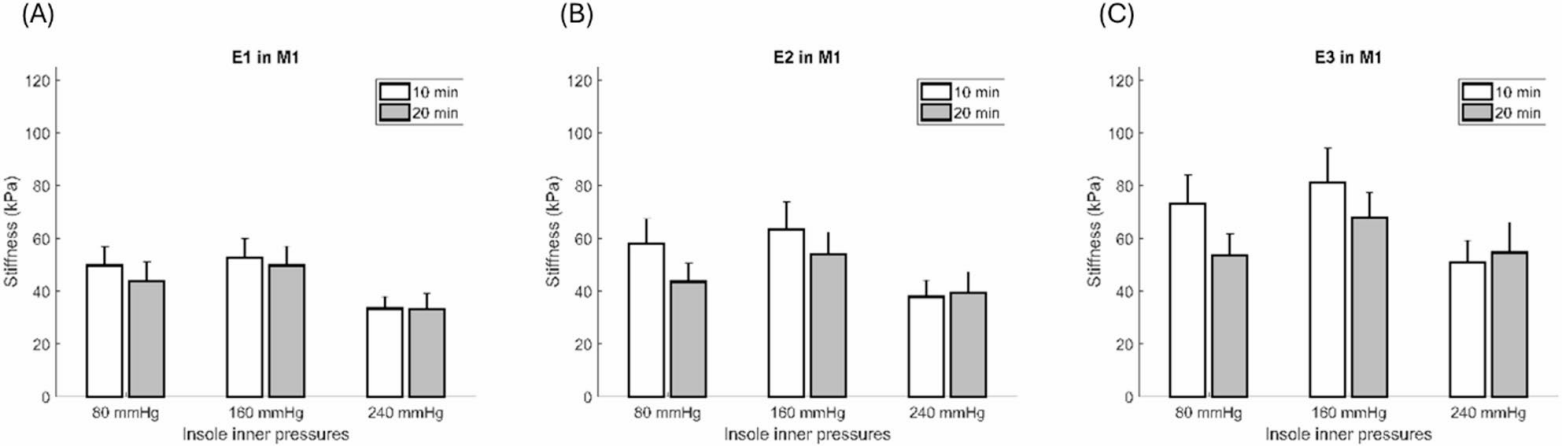
Comparisons of the effect of walking durations on the elasticity at three inner pressures of the insole. **A** Effect of walking durations on the elasticity in *E1*; (**B**) Effect of walking durations on the elasticity in *E2*; (**C**) Effect of walking durations on the elasticity in *E3*. Data are shown as mean ± standard errors. M1 = first metatarsal head, *E1* = Toe 5%, *E2* = Heel 10%, *E3* = Linear 15%

Data were analyzed from all 13 participants. Regarding the effect of walking duration, the paired t-test showed M1 tissue elasticity at 80, 160, and 240 mmHg, with no meaningful difference observed (Table 4; Fig. 6A and B, and 6C).

**Table 4:** Effect of walking duration on the elasticity

Parameter	Inner Pressure of Insole	Walking Duration		Paired t-test
		10 min (Mean ± SE)	20 min (Mean ± SE)	*P* value
*E1* (kPa)	80 mmHg	49.8±7.2	43.8±7.3	0.303
	160 mmHg	52.7±7.3	49.8±7.3	0.744
	240 mmHg	33.4±4.6	33.2±6.1	0.958
*E2* (kPa)	80 mmHg	58.2±9.2	43.6±7.0	0.074
	160 mmHg	63.5±10.3	54.1±8.4	0.448
	240 mmHg	37.9±6.3	39.5±7.9	0.655
*E3* (kPa)	80 mmHg	73.0±10.9	53.7±8.0	0.081
	160 mmHg	81.1±13.0	67.7±9.6	0.376
	240 mmHg	50.8±8.5	54.7±11.3	0.482

Data are presented as the mean ± standard error

*E1* = Toe 5%, *E2* = Heel 10%, *E3* = Linear 15%

**Fig. 7:**
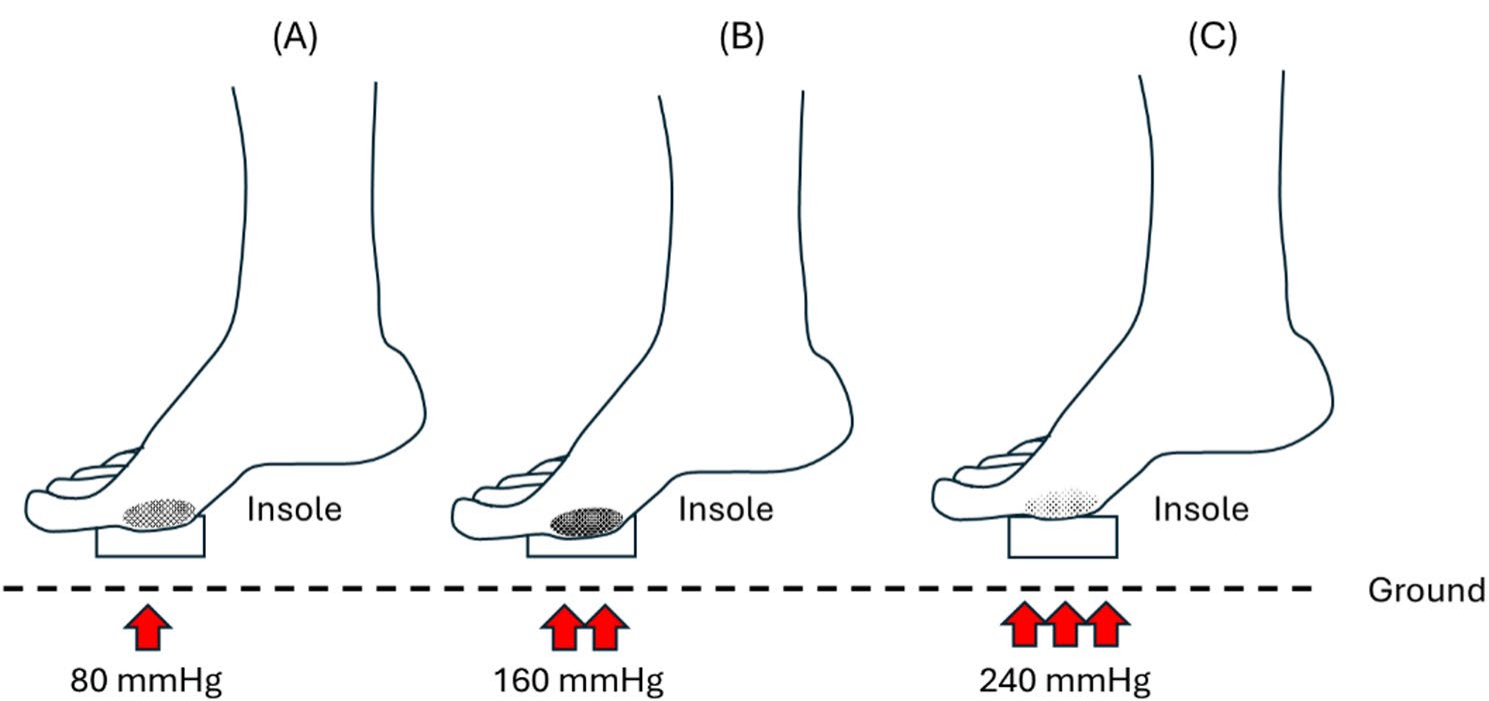
Illustration of different elasticity effects on the insole hardness. **A**. 80 mmHg insole hardness, medium stiffness; (**B**) 160 mmHg insole hardness, highest stiffness; (**C**) 240 mmHg insole hardness, lowest stiffness

The illustration below shows the difference in the effect of three insole hardnesses after walking exercise. And the lowest stiffness was obtained in the M1 region with the 240 mmHg insole hardness after walking (Fig. 7).

## Discussion

This study aimed to enhance our understanding of how inner pressure insoles and walking duration affect elasticity in the M1 region. *E1* and *E2* were significantly lower in stiffness at an inner pressure of 240 mmHg than at 160 mmHg. However, there was no significant difference in *E3* elasticity across all variations in the inner pressure insoles. Additionally, there was no significant difference in M1 elasticity between walking durations of 10 and 20 min. Furthermore, our results showed no significant difference in elasticity at 240 mmHg between 10 and 20 min walking durations.

Our first result showed that the 240 mmHg inner pressure insole had the lowest stiffness. According to previous studies, peak plantar pressure in people with diabetes results in greater forefoot deformities than in rearfoot deformities [45, 46]. Significantly, we aligned these findings with our study to measure M1 elasticity. When a repetitive load is applied, it releases pressure and triggers endothelial and muscle cells to increase vasodilation and vasodilatory functions, resulting in increased microcirculation [47]. The lower stiffness in the M1 region obtained with the 240 mmHg inner pressure insole indicates a consistent increase in microcirculation during the first 10 to 20 min of walking (Fig. 7). This could occur due to temporary fluid shifts, increased blood flow to the capillaries, and slight tissue swelling during early movement [48]. Still, this connection depends on the situation, and stiffness alone shouldn’t be taken as a sign of better or worse blood flow without directly measuring circulation.

In our results, *E1* and *E2* showed significant differences, but not in *E3*. The *E1* corresponds to the area of the curve where the x-axis is most linear, usually up to 0.3 strains [49]. In the initial phase of mechanical loading, collagen fibers’ wavy structure straightens, resulting in minimal resistance to applied stress. During this stage, elastic fibers direct the tissue’s mechanical response, allowing significant deformation under low stress levels. This behavior is characteristic of the toe region in stress-strain curves of soft connective tissues [50]. At *E2*, the slope of the curve increased. At *E2*, the slope of the curve increased. As a result of the applied stress, the curves in the gap regions of the fibrils begin to straighten out, and the fibrils begin to stretch and align in the direction of the applied stress [17]. At this stage of the stress-strain curve, collagen begins to resist deformation, requiring higher stress levels to maintain tissue deformation. We speculate that no meaningful difference in *E3* suggests that it serves as a more gradual transition between *E2*, characterized by high deformation, and *E1*, characterized by lower deformation, leading to a more linear and less variable response. In addition, it may be a linear transition zone where the mechanical properties and load responses are more balanced or uniform than those of the distinctly different *E1* and *E2* regions.

In addition, walking durations of 10 and 20 min showed no significant differences. However, according to our results, there was a trend that the 20 min walking duration had a lower stiffness than that of 10 min. Furthermore, low stiffness is associated with improved microcirculation, which ensures a better supply of nutrients and oxygen to the tissues, thereby promoting healthy tissue function and repair [26]. Impaired circulation may result in delayed healing and reduced elasticity in diabetic patients. Thus, maintaining adequate microcirculation helps prevent excessive crosslinking of collagen fibers, which may increase elasticity [27]. Based on our previous study, longer walking durations increase the reactivity of the plantar microcirculation owing to increased activity time, which may help reduce the risk of DFUs [51]. At the final 3 min post-exercise, the 20 min walks consistently enhanced microcirculation more than the 10 min. Notably, 10 min walking with 80 mmHg insole pressure led to significantly lower microcirculation than 240 mmHg as measured by a laser Doppler probe [51]. However, it is important to note that each participant used a unique dataset. Several factors can influence pressure parameters, including body weight, walking pattern, and foot deformity [52].

Regarding the use of the 240 mmHg insole hardness that resulted in the lowest stiffness, while it showed a significant difference in *E1* and *E2*, it did not show any significant difference between the 10 and 20 min durations. Using the 240 mmHg forefoot insole hardness may further compromise already weakened plantar tissue. It may lack sufficient structural support as the tissue becomes overly soft, resulting in excessive compression and concentrated pressure, which may increase the risk of ulcer formation. Clinically, this suggests that pressure distribution and insole design should be carefully optimized, avoiding high localized pressures that coincide with areas of lowest tissue stiffness and prevent DFU development [53]. In contrast, a walking duration of 10 min showed a higher trend in stiffness with 80 and 160 mmHg inner pressure insoles than that at 20 min. An ideal effective Young’s modulus (***E***) at the M1 for DFU prevention is likely in the ~ 40–60 kPa range, soft enough to provide cushioning while maintaining structural integrity. This range is consistent with values observed in healthy plantar tissue [37] and is consistent with clinical strategies aimed at reducing ulceration risk by promoting optimal load distribution through custom insoles or offloading devices. While there is no universally established plantar pressure threshold for preventing ulceration, optimizing the distribution of forces across the foot has been shown to reduce tissue stress and risk of injury [48]. Several studies offered insights that can guide the design of footwear interventions, complementing hydraulic system and removable insole systems that actively adapt to the wearer’s foot in intelligent footwear [54, 55].

Although this study has some strengths, it has some limitations. First, participants in this study were non-diabetics and generally younger than the typical recipient of orthotic interventions for preventing DFU [56]. It included only 13 healthy subjects, limiting the generalizability of the findings. Measurements were also conducted on separate days, which may confound the effects of insole pressure with inter-day variability. A larger sample size would provide more robust data and improve the reliability of results. Second, the current study examined only M1 elasticity but not other foot regions that may exhibit different pressure and duration responses. In addition, our device does not quantify the measurement error for tissue elasticity. Foot deformities in individuals with DM often occur due to motor neuropathy, leading to muscle atrophy and imbalance [57]. Previous studies have shown that modifying foot support, such as with wedges, can influence lower extremity muscle activity and delay fatigue during dynamic tasks [58–60]. Similarly, our results indicate that air insole conditions affect plantar soft tissue deformation, suggesting that optimized foot support may simultaneously reduce tissue strain and improve functional performance during walking. There is a critical need to understand the pressure response in these different plantar regions [61]. Therefore, future studies may be beneficial in determining walking thresholds and preventing complications in patients with DM. Third, the analysis of paired t-tests revealed that walking duration had no significant effect on elasticity at 10 and 20 min for the three different insole pressures. As shown in a study by Duan et al. 2021, the elasticity of the plantar surface is not significantly affected by daily activities or short-term variations in pressure [62]. Although people with DM are recommended to exercise for 10 min following the significant difference between *E1* and *E2* values, we did not study the long-term effects of repeated walking sessions or chronic exposure to high pressure. Understanding these long-term effects is crucial for practical applications and recommendations. Future studies could use a longer duration to provide more insights into the effects of sustained pressure on elasticity and extend this investigation to older adults and individuals with diabetes to explore patterns in tissue stiffness response and the effects of insole pressure modulation.

## Conclusion

The present study suggests that the inner pressure of the insole and walking duration significantly influence plantar elasticity, which can help prevent risks associated with DFU. Amongst all inner pressure insoles, the plantar elasticity at an inner pressure of 240 mmHg had the lowest values after both 10 min and 20 min of walking. All inner insole pressures showed a reduction in E1 and E2 tissue elasticity after 20 min of walking, except for E3. In addition, we found that the 240 mmHg insole pressure reduced stiffness beyond the recommended range for walking interventions. Therefore, this pressure level should be avoided, as walking exercise under such conditions may elevate the risk of excessive mechanical loading on the plantar soft tissues. By examining variables such as inner pressure insoles and walking duration, footwear interventions can be optimized.

## Data Availability

The authors confirm that the data supporting the findings of this study are available within the article.

## Abbreviations

DM: Diabetes Mellitus
DFU: Diabetic foot ulcer
E: Effect Young's Modulus
E1 (toe): 5% strain
E2 (heel): 10% strain
E3 (linear): 15% strain
M1: First metatarsal head
ANOVA: One–way analysis of variance
